# Helical Tomotherapy for Inoperable Breast Cancer: A New Promising Tool

**DOI:** 10.1155/2013/264306

**Published:** 2013-09-02

**Authors:** Ciprian Chira, Youlia M. Kirova, Xavier Liem, François Campana, Dominique Peurien, Malika Amessis, Nathalie Fournier-Bidoz, Jean-Yves Pierga, Rémi Dendale, Pierre Bey, Alain Fourquet

**Affiliations:** ^1^Department of Radiation Oncology, Institut Curie, 26 rue d'Ulm, 75005 Paris, France; ^2^Department of Medical Physics, Institut Curie, 26 rue d'Ulm, 75005 Paris, France; ^3^Department of Medical Oncology, Institut Curie, 26 rue d'Ulm, 75005 Paris, France

## Abstract

*Background*. We investigated the feasibility of helical tomotherapy (HT) for inoperable large breast tumors, after failing to achieve adequate treatment planning with conformal radiation techniques. *Material and Methods*. Five consecutive patients with locally advanced breast cancer (LABC) were treated by preoperative HT. All patients received up-front chemotherapy before HT. Irradiated volumes included breast and nodal areas (45–50 Gy) in 4 patients. One patient received a simultaneous integrated boost (55 Gy) to gross tumor volume (GTV) without lymph node irradiation. Acute toxicity was assessed with Common Toxicity Criteria for Adverse Events v.4. Patients were evaluated for surgery at the end of treatment. *Results*. Patients were staged IIB to IIIC (according to the AJCC staging system 2010). HT was associated in 4 patients with concomitant chemotherapy (5-fluorouracil and vinorelbine). Two patients were scored with grade 3 skin toxicity (had not completed HT) and one with grade 3 febrile neutropenia. One patient stopped HT with grade 2 skin toxicity. All patients were able to undergo mastectomy at a median interval of 43 days (31–52) from HT. Pathological partial response was seen in all patients. *Conclusions*. HT is feasible with acceptable toxicity profiles, potentially increased by chemotherapy. These preliminary results prompt us to consider a phase II study.

## 1. Introduction

Locally advanced breast cancer (LABC), defined mainly by stage III disease [1] and by a subset of stage IIB (T3N0), occurs in less than 15% of the diagnosed women [2–4] but poses a significant challenge from a treatment point of view. It requires a combined treatment approach involving anthracycline-based chemotherapy (with or without a taxane) and trastuzumab for human epidermal growth factor receptor 2 (HER-2) positive tumors, followed by surgery and radiation therapy [5]. But for patients with large volume disease whose tumors remain inoperable after primary or neoadjuvant chemotherapy (NCT) the management strategy is less clear. 

Recent studies have used preoperative radiotherapy (combined with chemotherapy) in an attempt to downsize the tumor [6–11] making it amenable to surgery. However, these studies have only used conventional radiation techniques with considerable limitations in target volume coverage and sparing normal tissues. 

**Figure 1:**
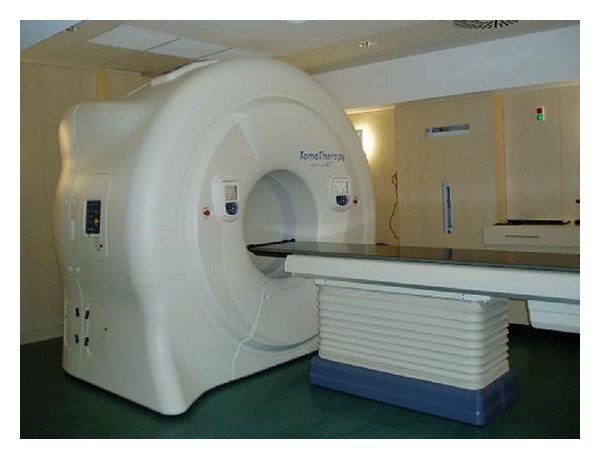
One of the two TomoTherapy Hi-Art treatment systems used in this study.

Helical Tomotherapy (HT) is a new form of intensity-modulated radiation therapy (IMRT) that delivers a modulated fan beam using a 6 MV linear accelerator mounted on a ring gantry that rotates around the patient as he/she advances slowly through the gantry bore (Figure 1). Its advantages include: ability to correct for set-up errors, delivery of continuous craniocaudal irradiation which suppresses junction problems, and the conformality of the dose distribution throughout the complex volumes formed by the lymph nodes and the breast [12].

We sought to report our early experience with the use of HT (with or without CCT) for inoperable LABC not eligible to conformal radiation techniques due to disease extension.

## 2. Patients and Methods 

From November 2007 to February 2011 five consecutive women with stage IIB–IIIC LABC (according to AJCC staging system 2010) were seen at our multidisciplinary clinic. All patients had histological confirmation of malignancy by tumor biopsy with determination of tumor oestrogen and progesterone receptor (ER/PR) status and HER-2. The workup included history and physical examination with recording of size and location of the tumor on a diagram of the affected breast and a photo evaluation. Adequate biology lab tests were undertaken. Imaging studies included bilateral mammogram and breast ultrasound or breast magnetic resonance imaging (MRI), bone scan, thoracic-abdominal and pelvic computed tomography scan (CT), and fluorodeoxyglucose (FDG) positron-emission tomography scan (PET/CT) in one case. Genetic counseling was necessary in one patient. 

**Figure 2:**
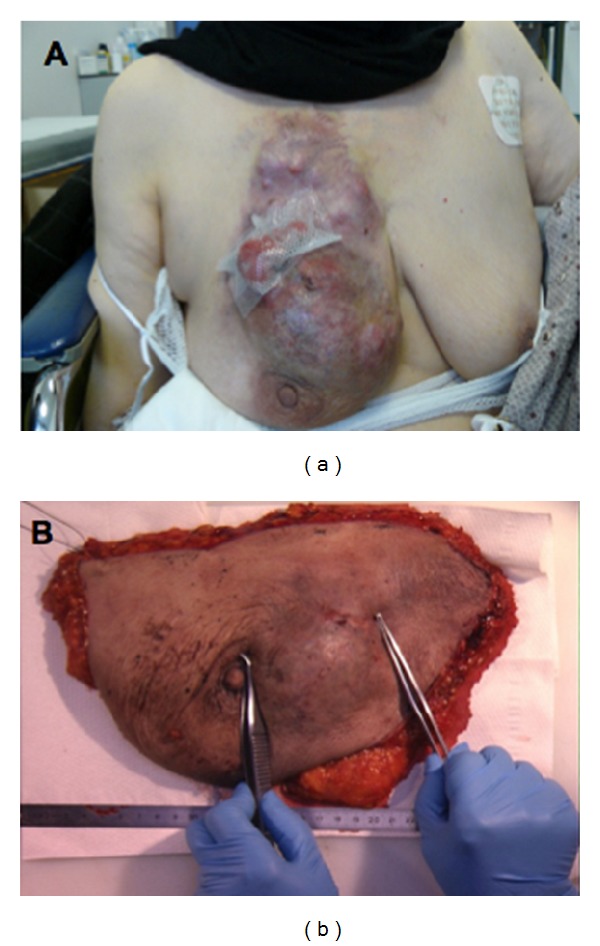
(a) Large breast tumor in one of our patients before initiation of treatment. (b) Macroscopic residual tumor (right image) on surgical specimen from the same patient.

All patients had advanced voluminous breast tumors judged not amenable to any form of surgery (conservative or radical). Inoperable breast cancer was defined as a combination of at least 2 of the following criteria (except for inflammatory breast carcinoma): fixation of the axillary nodes to overlying skin or deeper structures of the axilla, skin ulceration, inflammatory breast carcinoma, solid fixation of tumor to the chest wall, extensive edema of the skin (involving more than one-third of the skin over the breast), massive involvement of axillary lymph nodes (measuring 2.5 cm or more in transverse diameter), or clinically involved periclavicular lymph nodes and internal mammary metastases as evidenced by a parasternal tumor [13]. Resectability was evaluated by the breast surgeon based on the above criteria and available radiological imaging.  Figure 2 illustrates the clinical assessment of one of these patients. One patient presented with a large primary (T3N0), located in the upper inner quadrant, being considered inoperable due to low probability to achieve clear surgical margins.

All patients received up-front NCT before radiation delivery due to size and extent of disease and thus for the risk of micrometastatic disease. Chemotherapy regiments used before HT are detailed in Table 1. Clinical tumor response (defined at last week of NCT) was reported as complete if there was no palpable tumor in the breast, as partial if there was a reduction in tumor size (product of the two greatest perpendicular diameters) >50%, and as progressive disease when there was an increase >50%. Tumors not meeting these criteria were considered to be stable disease [14]. 

### 2.1. HT Planning and Radiation Delivery

In all 5 cases the choice of HT was done after careful dosimetry planning in three-dimensional conformal radiotherapy (3D CRT). An “optimized” 3D field-in-field technique, associated with internal mammary (IMN) electron-beam planning, was used, which is the current standard in our department [15]. Two tangential fields with superimposed posterior borders, matching supraclavicular (SCV) and IMN fields (when indicated) were generated. For each tangent, one subfield was created with the MLC shaped to shield the 107% isodose, and the other increased the dose in the thickest part of the breast, if necessary. A more comprehensive description of this planning procedure has been published elsewhere [16]. The dosimetrical analysis using 3D CRT showed in all cases inadequate target volume coverage and unacceptable high doses to some critical organs.

**Figure 3:**
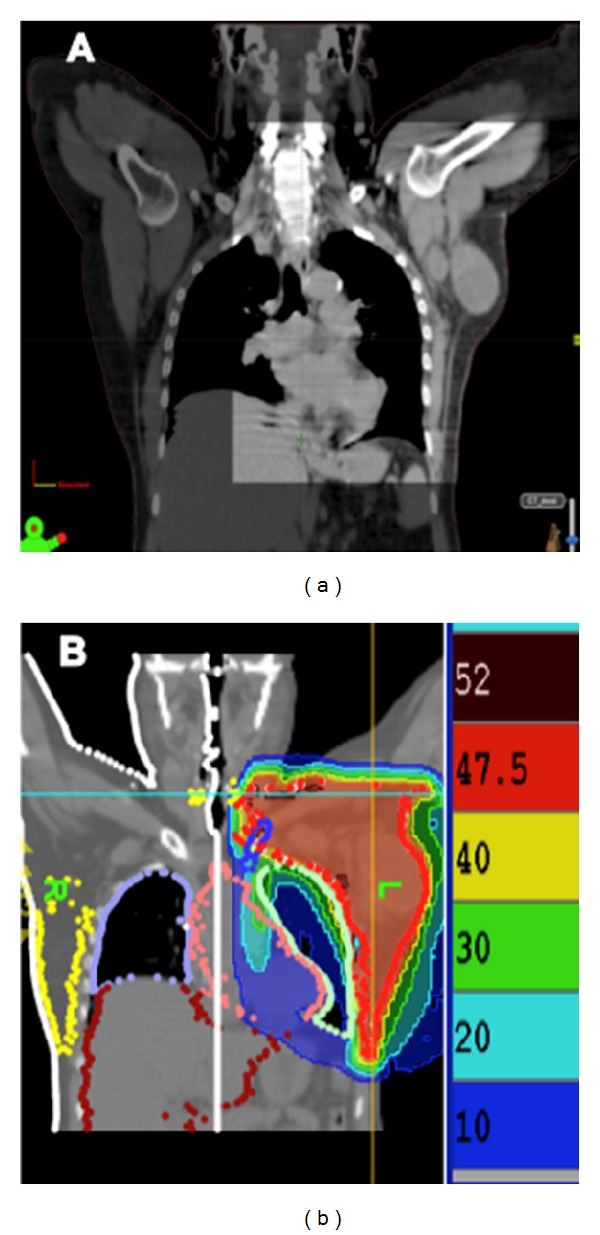
(a) Coronal view of planning CT scan. (b) Dose colorwash of helical tomotherapy (HT) treatment plan.

The treatment planning CT scan was performed 1-2 weeks after the last cycle of NCT. Patients were placed in the supine position, on a breast board, with both arms abducted alongside the head. The palpable breast tissue contour and the tumor were delineated with radioopaque wires. Radioopaque markers were also placed along the midsternum, as well as 1-2 cm below the palpable breast limits. Images were acquired from the upper neck to the midabdomen, using a 3 mm slice thickness and separation. The CT data were transferred to a commercial treatment planning system (Eclipse 3D version 8.1; Varian Medical Systems Inc., Palo Alto, USA). The breast clinical target volume (CTV) was defined as the tissue delineated by the aforementioned radioopaque wire. In practice, on each transverse slice, the breast volume extended from the pectoralis major muscle to the skin, excluding the pectoralis muscle, ribs, or the first 3 mm of skin except in inflammatory tumors. Breast planning target volume (PTV) was generated by adding a tridimensional margin of 5 mm around the breast CTV. The gross tumor volume (GTV) was defined on the planning CT as the tissue delineated by the radioopaque wire. Margins were then added to GTV based on the information from initial clinical and radiological reports (boost CTV). Boost PTV was defined adding an additional margin of 5 mm beyond boost CTV. However, a simultaneous integrated boost (SIB) was delivered in only one patient. The regional lymph nodes (axillary (ALN), internal mammary (IMN), supraclavicular (SCV)/infraclavicular (IFC)) were delineated (whenever indicated) using our atlases [17, 18]. The heart was contoured from the level of the pulmonary trunk to the apex and included the pericardium but not the major vessels. Lungs, spinal cord, contralateral breast, esophagus, and thyroid gland were also manually delineated (Figure 3). The CT data and the structure sets were transferred to the tomotherapy planning station (TomoTherapy Hi-Art version 3.1.2.3; TomoTherapy Inc., Madison, USA). All plans used a jaw width of 2.5 cm, a pitch of 0.286, and a modulation factor of 2.5. Two complete blocks were created on the treatment planning system to improve HT planning. Block 1 encompassed the whole contralateral breast and hemibody, while block 2 encompassed the posterior part of the ipsilateral side of the body. The initial DVH constraints and penalties are shown in Table 2. These were adjusted during optimization to obtain adequate target volume coverage while minimizing heart, lung, esophagus and thyroid irradiation. The aim was to achieve a full PTV coverage between 95% and 107% of the prescribed dose (with the 95% isodose set as the reference isodose), to attain high target-dose homogeneity, to minimize the volume of normal tissue that received a high dose, and to keep the dose to critical structures below their tolerance. For organs at risk (OARs), the dosimetric constraints were set according to previously published toxicity data, reviewed in the QUANTEC recommendations [19]. The heart volume that received 25 Gy was limited to 10% [20], and the 20 Gy volume of both lungs was limited to 30–35% [21]. Coverage was considered adequate when the aforementioned criterion was met. Furthermore, an effort was made to reduce the treatment volume receiving more than 107% of the dose to the tumor to less than 1%.

### 2.2. Concomitant Chemotherapy (CCT) Regimen Used in Combination with HT

Concomitant chemotherapy (CCT) consisted of 4 cycles of 5-fluorouracil (5-FU), 500 mg/m^2^/d, administered by continuous intravenous infusion over five consecutive days (d1–d5), and vinorelbine, 25 mg/m^2^, short intravenous infusion on days 1 and 6. Courses were repeated every 3 weeks for a total of four courses. Radiotherapy started on day one of the second course of chemotherapy. Two cycles were prescribed during radiotherapy. This CCT protocol was tested in our institution in a phase II trial and was previously published [22, 23]. 

### 2.3. Evaluations of Toxicity and Pathological Response

Patients were seen on a weekly basis during HT. All toxicities were graded according to the Common Terminology Criteria for Adverse Events (CTCAE) v.4 [24]. 

Pathological response assessment on surgical specimen took into account the proportion of residual tumor cells, the location of this malignant component (invasive versus intraductal), the mitotic index in malignant cells, and the status of the metastatic axillary nodes. The response was considered as pathologically complete (pCR) when there was no residual invasive malignant epithelial cells in both the breast and the axillary lymph nodes. Tumors with an epithelial malignant residual component strictly in situ or representing less than 5% of the breast and/or axillary tumor mass and without any mitosis were also classified in the group of pCR. The response was considered as absent (pSD) when no histological modification of the tumor tissue could be related to therapy and as partial (pPR) in the remaining cases. This is according to the interpretation at the Institut Curie of the definition proposed by Sataloff and colleagues of a “total or near total therapeutic effect” [25, 26]. 

## 3. Results

Patient and initial tumor characteristics are described in Table 1. All patients had invasive ductal adenocarcinoma and had good performance status (ECOG score 0-1). Most NCT regimens were taxane- and anthracycline-based regimens (Table 1). Median number of delivered cycles was 8 (range: 6–8). 

Planning with 3D CRT revealed that the doses to PTV did not attain the 95% constraint in 3 cases (<85%). Furthermore, mean (*D*_mean_) and maximum dose (*D*_max⁡_) as well as V20 constraints for ipsilateral lung were not achieved with 3D CRT in 4 patients. Equally, *D*_max⁡_ and V25 for the heart had not been achieved with 3D field-in-field technique in one patient with a left-sided large tumor.

Histogram dose-volume points were achieved with HT planning without deviation from the protocol for organs at risk (Table 2). Prescribed doses of radiation varied from 47.5 Gy in 25 daily fractions of 1.9 Gy to 50 Gy in 25 fractions, with simultaneous integrated boost to CTV of up to 55 Gy in 25 fractions of 2.2 Gy. Delivered doses are described in Table 3. 

There was no toxic death. Early grade 3 skin toxicity in the irradiated field was seen in 2 patients (patients numbers 4 and 5, Tables 3 and 4) both receiving CCT. These 2 patients required treatment interruption for skin care at 46 Gy/23 fx (planned dose was 50 Gy). The rest of patients experienced grade ≤2 skin events. Patient number 1 (Tables 3 and 4) had also stopped treatment at 41.8 Gy/22 fx (planned dose was 47.5 Gy/25 fx) while being scored with grade 2 skin toxicity, due to extent of lesions as well as patient desire. There was no grade >1 digestive toxicity. Grade 3 febrile neutropenia was observed in 1 patient (number 5 in Table 4). No cardiac or pulmonary toxicity was recorded during treatment and follow up.

Clinical evaluation of response to HT was judged favorable, and all patients were finally considered eligible for radical surgery. Modified radical mastectomy (MRM) with axillary lymph node dissection (ALND) of the first two levels was performed in all cases. Median time to surgery from last day of radiotherapy was 43 days (range: 31 to 52). Pathological response assessment on surgical specimen revealed pPR in all patients, according to the modified Sataloff criteria (Table 4). No patient achieved complete pathologic response.

Margins were negative in all cases (>0.7 cm in 4 cases, 5 mm in one case). No fibrosis was described in the surgical reports. One patient had wound infection and needed surgical drainage. Two patients had aspirations of lymphoceles. 

Adjuvant treatments were decided according to pathological criteria and consisted of either chemotherapy (absence of complete pathological response) and/or endocrine therapy (presence of positive expression of ER/PR).

Median follow up was 15.4 months (range: 2 to 25.1). At last follow up, 2 patients were still alive and free of disease, presently undergoing endocrine therapy. One patient was lost to follow up, and 2 patients had died from metastatic disease. 

## 4. Discussion

In the present study we have tested a relatively new form of radiation combined with sequential and/or concomitant chemotherapy. To the best of our knowledge this is the only exploratory study of HT in inoperable LABC. As it can be seen in Figure 2, these were patients requiring radiation treatment on extremely large and complex target volumes. 

HT appears to improve target coverage while sparing OAR because of its ability to achieve a higher degree of conformity to the PTV. The well-known ability of HT to treat breast cancer with complex treatment volumes [12] and regional lymph nodes [27, 28] has been published before. Unfortunately, these studies are difficult to compare because dosimetric reports have different aims and different clinical situations.

In the current study, we have seen that HT can significantly spare the ipsilateral lung (*D*_max⁡_ < 40 Gy) and reduce the lung V20 and V5 below tolerance levels. Wang et al. [29] showed the importance of the V5 which was a significant factor for the subsequent development of pneumonitis with a cut-off value of 42%. Therefore, the reduction of lung V20, V5, and mean lung dose is an important feature.

HT was also used in our series with the intention to avoid eventual cardiovascular toxicity, knowing that patients had previously received anthracycline (with or without bevacizumab) or taxane-based NCT. The reported rates of cardiac dysfunction vary from 4 to 7% in patients receiving Trastuzumab alone and up to 27% with concomitant trastuzumab, antracycline, and cyclophosphamid [30]. Epirubicine (used also in our study) is associated with 11.4% risk of cardiovascular toxicity [31]. The use of modern radiation techniques has been associated with a decline in cardiac mortality [32, 33]. In our patients, the HT plans resulted in acceptable doses to the heart. V25 Gy was negligible (<0.15 cc) with slight increase in *D*_mean_ compared to 3D CRT. Our results are consistent with other studies in which HT was tested in left-sided tumors with lymph node disease. Caudrelier et al. [28] also reported that cardiac dose was reduced with HT compared to 3D CRT (V30 Gy of 1.5% ± 1.9% versus 3.2% ± 2.2%). Their *D*_mean_ of the heart was 7.0 Gy (±2.9 Gy) versus 5.5 Gy ± 1.4 Gy (*P* = 0.2). Similar results were published by Goddu et al. [27] who reported a decrease in mean V35 Gy (from 5.6% ± 4.8% to 2.2% ± 1.5%) in the tomotherapy plans compared with 3D CRT. However, they showed an increase in *D*_mean_ to the heart compared to 3D CRT (12.2 ± 1.8 Gy versus 7.5 ± 3.4 Gy). 

The same protective cardiac feature of HT on the heart (from high doses) was also described by Coon and colleagues [34] in patients with unfavorable cardiac anatomy. In our study none of our 5 patients (2 left-sided) experienced cardiac dysfunction during follow up.

Regarding skin toxicity, our findings indicate that the rate of severe acute events (grade ≥ 3 CTCAE) is potentially increased by CCT, high radiation dose (>45 Gy/25 fx to lymph node volumes), and outspread of target volumes (breast only versus breast and lymph nodes). Doses of 50 Gy/25 fx to whole breast seem tolerable (without CCT or lymph node irradiation) with possibility of simultaneous boost to gross tumor volume (patient number 2, Tables 3 and 4). However, in treatment of both breast and lymph nodes (especially with CCT) doses should be limited to 45 Gy/25 fx (1.8 Gy/fx) to lymph nodes and 50 Gy/25 fx (2 Gy/fx) to whole breast. The toxicity of the above CCT regimen (combined 3D CRT) has been previously evaluated [23]. Nevertheless, this study is the first to report the acute toxicity of this CCT regimen combined with HT.

One of the most current challenges for radiation oncologists treating LABC patients is the field junction problem seen with irradiation of lymph nodes around the breast. In our cohort, 4 patients received HT irradiation of lymph node areas (except patient number 2 in Table 3). These patients were initially planned with conventional multiport techniques (CMT). From our experience we know that multiple adjacent fields can lead to either hot or cold spots in target areas. Even if solutions exist to overcome this problem (asymmetric jaws to create a half beam for SCV and IMN fields and couch rotations to align tangents to SCV/IMN fields), this adds complexity for the technologists during patients setup [35]. HT has not only the ability to correct setup errors but also the capacity to deliver a continuous craniocaudal delivery, which suppresses field junctions [36].

On the basis of the pathological analysis of surgical specimens our findings suggest that PR is achievable with HT and chemotherapy. Previous studies of LABC have reported good pathological response rates with preoperative chemoradiotherapy, but all used “conventional” radiation techniques, often via tangential fields. Matuschek et al. [11] reported a series of 315 LABC patients (cT1-cT4/cN0-N1). Preoperative EBRT delivered 50 Gy (5 × 2 Gy/week) to the whole breast, SCV/ICF nodes (255 of 315 patients), and IMC with a boost in 214 cases. Chemotherapy was administered prior to radiation in 192 patients and concomitantly in 113. Although pathologic complete tumor and nodal remission rate (pCR) was good (29.2%), in cT3 and cT4 patients it was significantly reduced (28% and 20%, resp.). Shanta and collegues [10] reported 1,117 consecutive LABC patients with stage IIB–IIIB (TNM staging, Heidelberg, Springer-Verlag; 1987) treated with neoadjuvant RT-CT (40 Gy/20 fx, 5 fx/week combined with CMF, EC, or AC). Complete pCR (pT0/pN0) was achieved in 33.7% of cases. While these studies indicate that high pCR can be achieved with conventional radiation techniques, detailed information on toxicity from these studies is scarce. Having in mind that LABC patients receive high doses of chemotherapy with potential toxicity and that conventional radiotherapy techniques have been associated with higher cardiac mortality [33], modern radiation techniques like HT should be examined. 

This study has some potential limitations that need to be considered. First, this is a retrospective study with a limited number of patients, and thus treatment results should considered with caution. However, LABC is quite rare and recruiting a significant number of patients is not easy. Second, pathological response rates to our treatment may be related to both NCT as well as HT combined or not with CCT. We acknowledge the crucial role of chemotherapy in locoregional control of LABC. In fact, as mentioned before, we have previously studied the role of preoperative chemo-radiation with the CCT regimen used in this study. We showed that pathological control rates are high even with the use of conventional radiation techniques. The purpose of this study was not to assess the impact of the HT on pathological response rates but rather to test the feasibility of HT in these complex cases. Finally, breast tomotherapy needs human resources for the preparation and delivery of treatment (contouring of all target and organs-at-risk volumes, dosimetry optimization, and quality controls). Thus, small community centers may not have sufficient financial resources for HT or human personnel for the HT workload.

## 5. Conclusion

Preoperative HT with or without CCT appears to be a feasible and promising alternative to highly conformal techniques in the treatment of large inoperable breast cancers. Particular attention should be given to evaluate acute skin toxicity especially in patients receiving CCT. Larger studies are warranted to better define HT doses and to evaluate long-term toxicities.

## Figures and Tables

**Table 1:** Patient and tumor characteristics.

Characteristic	Value
Age Median (range)	62 (28–65)
Clinical Stage*	
IIB	1
IIIA	1
IIIB-IIIC	3
Tumor diameter in mm Median (range)	88 (75–160)
Laterality	
Right sided	3
Left sided	2
Hormonal receptors and HER2 over-expression	
ER−, PR−, HER2−	2
ER+, PR−, HER2−	1
ER+, PR+, HER2−	1
ER−, PR−, HER2+	1
Histological grade^§^	
2	1
3	4
Number of mitoses/10 high power field	
<11	1
>22	4
Initial chemotherapy regimen before HT	
EC + docetaxel	3
FEC + docetaxel	1
Docetaxel + trastuzumab	1
Adjuvant hormonotherapy	
Yes	2
No	3

Abbreviations: ER: oestrogen receptor, PR: progesterone receptor, HER2: Human Epidermal Growth Factor Receptor 2, *AJCC Cancer Staging Manual, Seventh Edition (2010), EC: epirubicin, cyclophosphamide, FEC: 5fluorouracil, epirubicin, cyclophosphamide, ^§^Elston-Ellis modification of Scarff-Bloom-Richardson grading system.

**Table 2:** Parameters for organs at risk (OAR) during HT planning.

OAR	Priority	Blocking	Importance	Histogram dose-volume points
Contralateral lung	1	Directional	1000	5%-7 Gy
				30%-3 Gy
				50%-2 Gy
Heart	2	Directional	1000	15%-10 Gy
				5%-15 Gy
Homolateral lung	3	Directional	1000	50%-5 Gy
				15%-20 Gy
				5%-30 Gy
Contralateral breast	4	Directional	1000	10%-3 Gy
Spinal cord	5	Directional	300	30%-10 Gy
Liver	6	Directional	300	20%-5 Gy

**Table 3:** Description of treatment volumes and prescribed radiation doses with helical tomotherapy.

Patient number	Total doses (Gy)					Dose per fraction (Gy)				
	WB	Lymph nodes			TB	WB	Lymph nodes			TB
		IMN	SCV IFC	ALN			IMN	SCV IFV	ALN	
1	41.8	41.8	41.8	41.8	41.8	1.9	1.9	1.9	1.9	1.9
2	50				55	2				2.2
3	50		45	45	50	2		1.8	1.8	2
4	46	46	46	46	46	2	2	2	2	2
5	46	46	46	46	46	2	2	2	2	2

WB: whole breast, IMLN: ipsilateral internal mammary lymph nodes, SCV: ipsilateral supraclavicular fossa, IFC: ipsilateral infraclavicular fossa (level III axillary), ALN: ipsilateral level I and II axillary lymph nodes, TB: tumoral bed.

**Table 4:** Treatment characteristics and results.

Patient number	TNM stage^*∬*^	Tumor maximal diameter^†^ (mm)	WB dose^¶^ (Gy)	CCT/number of cycles	Early toxicity grade (CTCAE v.4)			Surgical specimen		Pathological response^§^
					Skin	Digestive	Other^‡^	T* size (cm)	Nodal status	
1	T4bN2aM0	105	41.8	Yes/4	2	0	0	50	7+/11	PR
2	T4cN2aM0	160	50	No	1	1	0	64	0/13	PR
3	T3N0M0	75	50	Yes/4	2	0	1	22	0/15	PR
4	T4bN2aM0	85	46	Yes/4	3	1	0	4.5	2+/8	PR
5	T3N2bM0	88	46	Yes/2	3	0	3	17.6	1+/9	PR

^∬^AJCC cancer staging manual, seventh edition (2010), WB: whole breast, CCT: concomitant chemotherapy, CTCAE: Common Toxicity Criteria for Adverse Events v.4, ^†^baseline evaluation before all treatments, ^¶^delivered radiation dose, ^‡^cardiovascular and/or pulmonary and/or hematological toxicity, *residual invasive malignant epithelial cells, ^§^interpretation at the Institut Curie of the concept proposed by Sataloff and colleagues (details in article), PR: partial response.
